# A Wearable, Textile-Based Polyacrylate Imprinted Electrochemical Sensor for Cortisol Detection in Sweat

**DOI:** 10.3390/bios12100854

**Published:** 2022-10-10

**Authors:** Samuel M. Mugo, Weihao Lu, Scott Robertson

**Affiliations:** Physical Sciences Department, MacEwan University, Edmonton, AB T5J4S2, Canada

**Keywords:** wearable capacitive sensor, cortisol sensing, molecularly imprinted polymers (MIPs), polyacrylate, e-skins

## Abstract

A wearable, textile-based molecularly imprinted polymer (MIP) electrochemical sensor for cortisol detection in human sweat has been demonstrated. The wearable cortisol sensor was fabricated via layer-by-layer assembly (LbL) on a flexible cotton textile substrate coated with a conductive nanoporous carbon nanotube/cellulose nanocrystal (CNT/CNC) composite suspension, conductive polyaniline (PANI), and a selective cortisol-imprinted poly(glycidylmethacrylate-co-ethylene glycol dimethacrylate) (poly(GMA-co-EGDMA)) decorated with gold nanoparticles (AuNPs), or plated with gold. The cortisol sensor rapidly (<2 min) responded to 9.8–49.5 ng/mL of cortisol, with an average relative standard deviation (%RSD) of 6.4% across the dynamic range, indicating excellent precision. The cortisol sensor yielded an excellent limit of detection (LOD) of 8.00 ng/mL, which is within the typical physiological levels in human sweat. A single cortisol sensor patch could be reused 15 times over a 30-day period with no loss in performance, attesting to excellent reusability. The cortisol sensor patch was successfully verified for use in quantification of cortisol levels in human sweat.

## 1. Introduction

Driven by a growing need for real-time data monitoring and decision making in personalized health & wellness monitoring, wearable sensor analytics platforms (e-skins) are in high demand [1,2,3]. Their low cost, portability, user-friendliness, and rapid response times contribute to their superiority over traditional analytical instrumentation [4]. Conventionally, blood and urine are extracted for diagnostics, which involves invasive sampling procedures and are not ideal for real-time analysis [5,6,7,8]. Interstitial fluid and sweat are rich in multiple biochemical metabolites, and are the most adaptable peripheral biofluids for diagnostics using wearable devices [4,5]. The wearable fitness technology market was estimated to be $45.5 billion in 2019, and it is expected to expand at a growth rate of 74% by 2026, likely to be accelerated by the COVID-19 pandemic [9,10]. To control the spread of the 2020 COVID-19 pandemic, governments in nearly all countries instituted strict travel restrictions, quarantines, and closure of workplaces, schools, and recreational activities, leading to social isolation. As such, the world’s population is on the verge of a mental health pandemic, with 70% of respondents in 63 countries reporting higher than moderate levels of depression [11,12,13,14]. To improve the outcomes for intervention in the growing mental health pandemic, deployment of wearable sensors for monitoring emotional health is a practical solution.

Cortisol is a well-known glucocorticoid hormone that is vital in physiological processes such as metabolism, electrolytic balance, and blood pressure regulation, all of which influence cognitive processes including working memory, sleep patterns, and mood [15,16,17]. Cortisol has also been recognized as a key biomarker of psychosocial stress, anxiety, depression, and mental health [18]. Physiological levels of cortisol in saliva, interstitial fluid, sweat, and blood generally fluctuate during circadian rhythm, peaking in the morning and decreasing throughout the day [19,20]. Levels of cortisol in human sweat can range from 0.24–144 ng/mL [16].

Most traditional cortisol detection methods, such as chemiluminescence immunoassays, capillary electrophoresis, GC-MS (LOD~0.72 ng/mL), and HPLC-MS (LOD~0.04 ng/mL), are lab-localized, time consuming, have complex sample preparation, and are therefore non-amenable for real-time monitoring [16,21,22,23,24]. Rapid and real-time monitoring is essential due to the natural diurnal fluctuations of cortisol levels. While portable sensor devices based on enzyme-tagging, antibodies, and DNA aptamer-based techniques offer excellent selectivity, they are environmentally unstable and suffer from poor reusability [25,26,27]. Molecular imprinted polymers (MIPs) are versatile, inexpensive, and thermochemically stable artificial molecular receptors with high affinity for binding their target molecules [28]. MIPs are ideal for designing stable biomimetic synthetic probes for cortisol detection. 

Previously, we have reported cortisol-specific polyacrylate-based MIPs printed on a conductive polydimethylsiloxane (PDMS) composite [21]. We demonstrate herewith the increased film stability and reusability of a polyacrylate MIP@PANI@CNT/CNC when coated on a cotton textile substrate, a flexible platform that lends to the uneven contours of the skin, thus making it adaptable as a wearable sensor. The anhydro-β-cellobiose units of the textile, along with increased surface area from the microporous microfibrous structure would afford effective loading of PANI, CNT/CNC, and the cortisol-imprinted polyacrylate, thereby increasing sensor sensitivity [29]. In addition, the cotton textile is an effective platform for high sweat sampling load. This article demonstrates the LbL assembly of a cotton textile substrate subsequently coated with a conductive, nanoporous PANI@CNT/CNC layer, and a cortisol-selective imprinted poly(glycidylmethacryate-co-ethylene glycol dimethacrylate) (poly(GMA-co-EGDMA)) polymer decorated with AuNPs (AuNPs@MIP@PANI@CNT/CNC@textile). The CNT/CNC, PANI, and AuNP layers were added to improve the electrical conductivity of the textile substrate, while the cortisol-imprinted poly(GMA-co-EGDMA) layer ensures selective capture of the cortisol analyte for detection. The cortisol sensor patch is 1.0 × 2.0 cm^2^ in size and 1–2 mm thickness, with screen printed counter and reference electrodes integrated onto the sensor platform.

## 2. Materials and Methods

The carboxylic acid functionalized multi-walled CNTs (OD: 4–6 nm. 98% pure) was purchased from TimesNano, Chengdu, China. Potassium ferricyanide (K_3_[Fe(CN)_6_]), ammonium peroxydisulfate (APS), 4,4′-azobis(4-cyanovaleric acid) (ACVA), acetonitrile, sulfuric acid, hydrocortisone, glycidylmethacrylate (GMA), gold nanoparticles (AuNPs) (5 nm) in citrate buffer, silver nanoparticles (AgNPs), sodium hypochlorite, ethylene glycol dimethacrylate (EGDMA), and cyclohexanol were procured from Sigma Aldrich, Oakville, Ontario, Canada. Aniline, dipotassium phosphate, and monopotassium phosphate were bought from Fisher Scientific, Hampton, NH, USA. Cellulose nanocrystals (CNC) were donated by Alberta Innovates, Edmonton, AB, Canada. 8330D conductive silver epoxy adhesive was purchased from MG Chemicals, Burlington, ON, Canada. The cotton bandage roll and acrylic sheets were purchased from a local grocery store. All reagents were of analytical reagent grade. All aqueous solutions were prepared using >18 MW Milli-Q deionized (DI) water.

### 2.1. Fabrication of AuNPs@MIP@PANI@CNT/CNC@Textile Cortisol Sensor Patch

To fabricate the AuNPs@MIP@PANI@CNT/CNC@textile cortisol sensor, an 8.0 × 8.0 cm^2^ cotton textile patch was evenly loaded with 10 mL of a CNT/CNC (1 mg/mL/4 mg/mL) homogenous suspension (in DI water), and air dried. This was followed by the loading of 6 mL of 0.1 M aniline dissolved in 0.5 M H_2_SO_4_, with 0.08 mL of 0.05 M APS added as the initiator, and polymerization allowed to ensue at 4 °C [30]. The PANI@CNT/CNC@textile was then cut into 1.0 × 2.0 cm size sensor patches. The PANI@CNT/CNC@textile sensor patch was then loaded with 100 µL of MIP prepolymer solution and allowed to polymerize in an oven at 70 °C for 4 h. The MIP prepolymer solution was comprised of 400 μL cyclohexanol, 26 μL GMA, 80 μL EGDMA, 2.0 mg ACVA, and 52 μL of 1.5 M of cortisol (dissolved in 1:1 (*v/v*) water: acetonitrile mixture). A control, non-imprinted polymer (NIP) sensor patch was fabricated using a similar procedure, but the prepolymer solution did not contain any cortisol template. Following polymerization, the cortisol from the MIP was electrochemically removed using cyclic voltammetry (CV) [21]. The CV was acquired using Palmsens 4 potentiostat with PSTrace software (PalmSens BV, Houten, The Netherlands). The MIP@PANI@CNT/CNC@textile cortisol sensor patches were electrochemically cleaned by immersion in 10 mL of 0.1 M phosphate buffer (pH = 7) in a conventional electrochemical cell comprising a platinum counter electrode and an in-house Ag/AgCl needle reference electrode. The reference electrode was prepared as described previously [31]. To ensure complete removal of cortisol, 45 CV cycles were applied (−1.0–1.0 V range, 0.1 V/s scan rate), and the phosphate buffer was replaced every 15 cycles. Patches were considered clean when the CVs taken during cleaning overlap and stabilized around a baseline (Figure S1). To enhance the conductivity of the cortisol sensor patch, 50 µL of AuNPs was added evenly onto the MIP@PANI@CNT/CNC@textile patch dropwise to yield the AuNPs@MIP@PANI@CNT/CNC@textile cortisol sensor patch (Figure S2).

**Figure 1:**
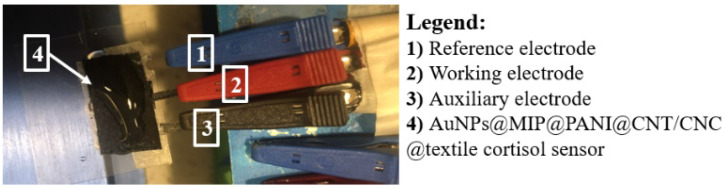
Camera image of the AuNPs@MIP@PANI@CNT/CNC@textile cortisol sensor patch and electrode orientation.

To ensure the use of the cortisol sensor in wearable applications, screen printed reference and auxiliary electrode were integrated into the sensor [32]. To make the in-house printed reference electrode, 10 mg of AgNPs were bleached overnight with 1 mL of sodium hypochlorite and air dried; followed by resuspension in 1 mL of CNT/CNC (1 mg/mL/4 mg/mL) in DI water. A PVA transparency sheet (0.2 × 1.0 cm^2^) was then evenly coated with 20 µL of the bleached AgNPs/CNT/CNC composite suspension, and allowed to air dry at room temperature. The auxiliary electrode was similarly prepared by coating a 0.2 × 1.0 cm^2^ PVA transparency strip with 20 µL of a CNT/CNC (1 mg/mL/4 mg/mL) suspension, and air drying at room temperature; followed by the addition of 10 µL of AuNPs (5 nm) in citrate buffer. The in-house reference and auxiliary electrodes were glued in proximity to the AuNPs@MIP@PANI@CNT/CNC@textile cortisol sensor patch (Figure 1). Au-plated MIP or NIP@PANI@CNT/CNC@textile patches were fabricated by sputter deposition of a 20 nm thin layer of gold onto a PANI@CNT/CNC@textile patch in this sensor configuration prior to the addition of the MIP/NIP layer and reference electrodes.

### 2.2. LbL Characterization of AuNPs@MIP@PANI@CNT/CNC@Textile Cortisol Sensor Patch

Fourier transform infrared spectroscopy (FTIR), scanning electron microscopy (SEM), electrochemical impedance spectroscopy (EIS), and CV techniques were used to characterize the AuNPs@MIP@PANI@CNT/CNC@textile cortisol sensor patch during LbL assembly. FTIR spectra were recorded using a Bruker Tensor 27 FTIR instrument fitted with diamond attenuated total reflectance (ATR). SEM images were acquired from a Zeiss Sigma 300 VP field emission SEM. EIS was performed by immersing the different patches in 25 mM K_3_[Fe(CN)_6_] (in 0.1 M KCl) as a standard redox probe in a three-electrode electrochemical cell, with an in-house fabricated Ag/AgCl reference electrode [31], and a platinum auxiliary electrode. To enhance electrical connectivity to the patch, a platinum electrode was attached to each textile patch with approximately 0.049 g of 8330D conductive silver epoxy glue (mixed in a 1:1 *v/v* ratio) and used as the working electrode. EIS data was acquired in the frequency range of 100 mHz–100 kHz at 10 mV of sinusoidal amplitude (vs. OCP) using a Palmsens 4 potentiostat with PSTrace software. CV analysis was used to determine the electroactive surface areas of the different patches, and was performed by immersing the patches in 5 mM K_3_[Fe(CN)_6_] (in 0.1 M KCl) in a three-electrode electrochemical cell, using an Ag/AgCl reference electrode [31], and a platinum auxiliary electrode. 

### 2.3. LbL Determination of Cortisol Sensor Patch Water Retention Abilities 

The water retention abilities of the cortisol sensor patches during its various stages of development was examined via a basic gravimetric test. The pre-weighed textile patch was immersed in DI water for 10 min, and excess water allowed to drip off the patch before reweighing. Water retention was calculated by subtracting the mass of the patch before and after immersion in water.

### 2.4. Voltammetric Testing of Cortisol Sensor Patches 

The cortisol textile sensor was evaluated for its electrochemical response to cortisol standards (9.8–49.5 ng/mL) using CV (−1.0–1.0 V, 0.1 V/s scan rate) acquired from a Palmsens 4 potentiostat with PSTrace software. This was done by adding a 300 µL aliquot of 0.1 M phosphate buffer (pH 7.1) on the cortisol sensor patch for the blank measurement, followed by 10 additions of 20 µL aliquots of 75 ng/mL cortisol, and then 5 additions of 20 µL aliquots of 150 ng/mL cortisol standards, with triplicate CVs acquired after each standard addition, 2 min of equilibration time were allowed prior to CV measurement. For each standard addition, three different textile sensors were evaluated and the response averaged to derive the calibration plot. The cortisol standard was prepared by dissolving hydroxycortisone in a 1:1 (*v/v*) mixture of DI water:acetonitrile. The faradaic capacitance was determined from the CVs by dividing the average cathodic current within a specified potential range by the CV scan rate. The Δcapacitance signal was determined by taking the ratio of the difference in sample and blank faradaic capacitance with blank capacitance. 

For the selectivity test, the Δcapacitance response of a MIP@PANI@CNT/CNC@textile cortisol sensor patch to lactate, ascorbic acid, estriol, and cortisol was determined. For each analyte, 400 µL of 0.1 M phosphate buffer was added onto the patch, and blank CV acquisition was acquired. Then, an aliquot of analyte was added onto the buffer-soaked patch, and CV was acquired after 2 min of equilibration. To clean the MIP@PANI@CNT/CNC@textile cortisol sensor patch for analysis of the next analyte, 500 µL of phosphate buffer was added to the sensor surface, and 15 CV cycles were then performed for electrochemical cleaning. Then, the sensor surface was rinsed with 4 mL of DI water, and completely dried in a dehydrator at 60 °C.

### 2.5. Sweat Analysis Using AuNPs@MIP@PANI@CNT/CNC@Textile Cortisol Sensor Patch

The cortisol textile sensor was further tested for the detection of cortisol in a real sweat sample collected from the brow of a volunteer after vigorous exercise. The standard addition method was employed for analysis, where a 200 μL aliquot of 0.1 M KCl was first deposited onto the AuNPs@MIP@PANI@CNT/CNC@textile cortisol sensor patch for blank CV acquisition. Then, a 100 μL aliquot of sweat sample was added onto the blank aliquot, followed by five 20 μL aliquots of 75 ng/mL cortisol standard, with CV being acquired in triplicate following each addition. To ensure reproducibility three textile sensors were evaluated, with each sensor used to analyzed each standard spiked sweat sample. Prior to CV measurements, the AuNPs@MIP@PANI@CNT/CNC@textile cortisol sensor patch was allowed to equilibrate for 2 min after each new addition. 

## 3. Results and Discussion

### 3.1. Stage-wise Characterization of MIP@PANI@CNT/CNC@Textile Cortisol Sensor Patch 

**Figure 2:**
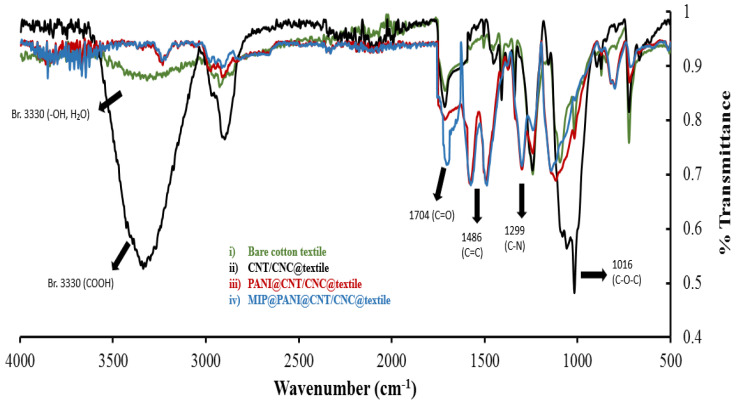
Overlapped FTIR spectra for (i) bare cotton textile; (ii) CNT/CNC@textile; (iii) PANI@CNT/CNC@textile; and (iv) MIP@PANI@CNT/CNC@textile patches.

The functional group signature of each stage of the cortisol sensor patches LbL assembly was determined by acquiring FTIR following addition of each layer on the textile substrate (Figure 2). The bare cotton textile shows a broad peak around 3330 cm^−1^, attributed to the hydroxyl groups of cellulose, lignin, and water; while the intense peak at 1016 cm^−1^ may be assigned to the vibrations of the C–O–C linkages in the pyranose ring of cellulose (Figure 2) [33,34]. Addition of the CNT/CNC film resulted in a characteristic intense broad peak at 3330 cm^−1^ (Figure 2), attributed to carboxyl groups of the modified multiwalled CNTs [35]. Both carboxyl and hydroxyl peaks decreased in intensity with subsequent addition of the PANI and cortisol MIP films (Figure 2). The PANI@CNT/CNC@textile patch had characteristic peaks at 1299 and 1486 cm^−1^ (Figure 2), attributed to the C–N stretching and C=C stretching of benzenoid and quinoid rings of PANI [32]. Addition of the freshly cortisol-imprinted poly(GMA-co-EGDMA) layer to the MIP@PANI@CNT/CNC@textile patch resulted in a characteristic band at 1704 cm^−1^ for C=O stretching vibration (Figure 2) [36].

**Figure 3:**
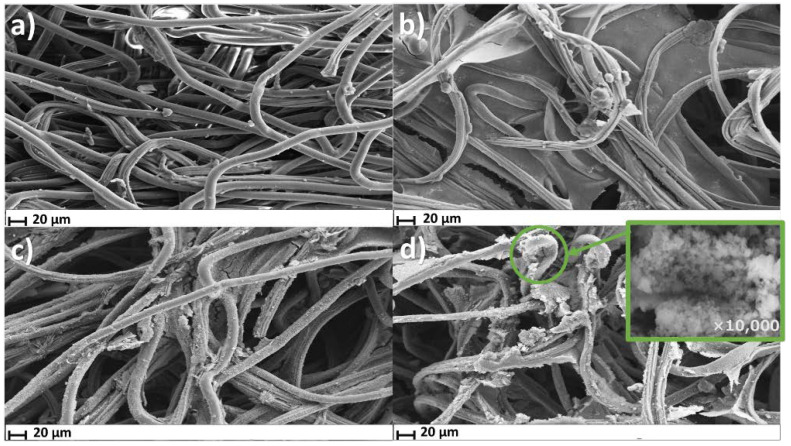
SEM images of (**a**) bare cotton textile; (**b**) CNT/CNC@textile; (**c**) PANI@CNT/CNC@textile; and (**d**) Cortisol-imprinted MIP@PANI@CNT/CNC@textile patches.

Following FTIR analysis, the surface morphology of the cortisol sensor patch during its LbL assembly was examined using SEM. Figure 3a,b show the morphology of a bare cotton textile patch and CNT/CNC@textile patch, respectively, with clear evidence of the CNT/CNC filling the nanoporous cellulose network of the cotton patch. Figure 3c shows the change in surface morphology upon addition of the PANI layer, evident by a characteristic rough and crystal-like coating. The PANI layer coated both the interstitial network filled with CNT/CNC, as well as the cotton fibers (Figure 3c). Figure 3d shows the surface morphology of the MIP@PANI@CNT/CNC@textile cortisol sensor patch, with a clear evidence of the increase in the patch surface roughness due to MIP formation. The inset figure in Figure 3d show the MIP layer in higher magnification with a clear evidence of the formation of the nanoporous monolithic structure characteristic of polyacrylate based polymers [21]. Mechanically, the AuNPs@MIP@PANI@CNT/CNC@textile cortisol sensor is flexible, and is able to be bent and crumpled without any significant damage (Figure S3).

The water retention abilities of the cortisol sensor patch during each stage of LbL assembly was evaluated using a gravimetric test. The water retention ability was determined to be 0.31 ± 0.03, 0.35 ± 0.05, 0.38 ± 0.05, 0.15 ± 0.03 and 0.17 ± 0.03 g/cm^2^ for the bare cotton textile, CNT/CNC@textile, PANI@CNT/CNC@textile, NIP@PANI@CNT/CNC@textile and MIP@PANI@CNT/CNC@textile patches, respectively. This demonstrates that the nanoporous cavity structure formed by addition of CNT/CNC and PANI to the textile patch increases the water retention ability of the textile sensor, while the addition of the MIP/NIP layer increases the hydrophobicity of the cortisol sensor patch, thus hindering the water retention ability. 

**Figure 4:**
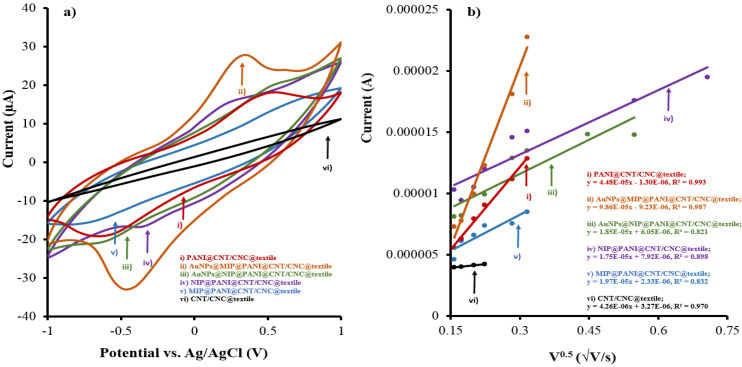
(**a**) Overlaid cyclic voltammograms; (**b**) resulting linear regression plot of peak current vs. square root of CV scan rate for (i) PANI@CNT/CNC@textile; (ii) AuNPs@MIP@PANI@CNT/CNC@textile; (iii) AuNPs@NIP@PANI@CNT/CNC@textile; (iv) NIP@PANI@CNT/CNC@textile; (v) MIP@PANI@CNT/CNC@textile; and (vi) CNT/CNC@textile patches.

The electroactive surface areas of the cortisol sensor patches at each step of the LbL assembly process was determined by running CV in 5 mM K_3_Fe(CN)_6_ (in 0.1 M KCl) at different scan rates, and invoking the Randles–Sevcik equation [37]. Figure 4a,b show the representative overlapped CVs and linear profiles for the cathodic peak current as a function of the square root of scan rates for textile sensors at different stages of their LbL assembly, respectively. Using the Randles–Sevcik equation for the cathodic current [37], the electroactive surface areas for each patch was determined, and is shown in Table 1. Addition of AuNPs to the MIP@PANI@CNT/CNC@textile patch resulted in an electroactive surface area enhancement of 399 %. Additionally, the slightly higher electroactive surface area of the MIP@PANI@CNT/CNC@textile cortisol sensor patch compared to the NIP@PANI@CNT/CNC@textile patch (Table 1) confirms the increased porosity due to the cortisol cavities present in the MIP platform following electrochemical cleaning [25].

**Figure 5:**
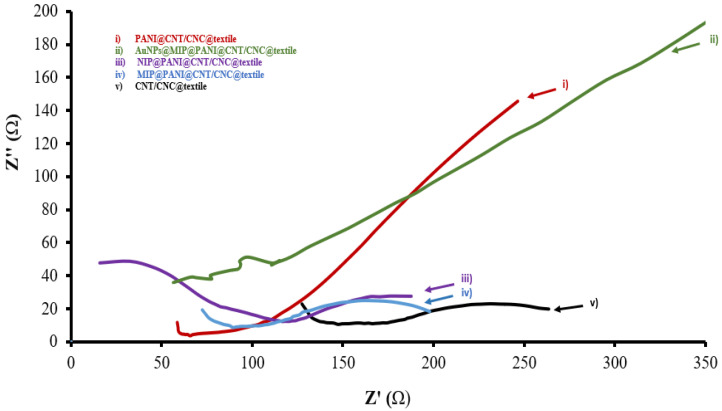
Overlapped Nyquist plots for (i) PANI@CNT/CNC@textile; (ii) AuNPs@MIP@PANI@CNT/CNC@textile; (iii) NIP@PANI@CNT/CNC@textile; 9 (iv) MIP@PANI@CNT/CNC@textile; and (v) CNT/CNC@textile patches.

In addition to CV, EIS was also used to characterize the electrical properties of the cortisol sensor patches at the different stages of LbL assembly, and the resulting overlaid Nyquist plots from analysis in 25 mM K_3_Fe(CN)_6_ solution (in 0.1 M KCl) for each patch are shown in Figure 5. The electron transfer resistance (R_ct_) extracted from the corresponding circuit fittings for each patch (Figure S4) are shown in Table 1. Addition of AuNPs to the MIP@PANI@CNT/CNC@textile patch reduced its electron transfer resistance by 28.7 %. Additionally, the PANI@CNT/CNC@textile patch had the lowest R_ct_, due to the increase in conductivity from interaction between the CNTs and PANI. 

### 3.2. Performance Evaluation of the MIP Cortisol Sensor and NIP Patches

**Figure 6:**
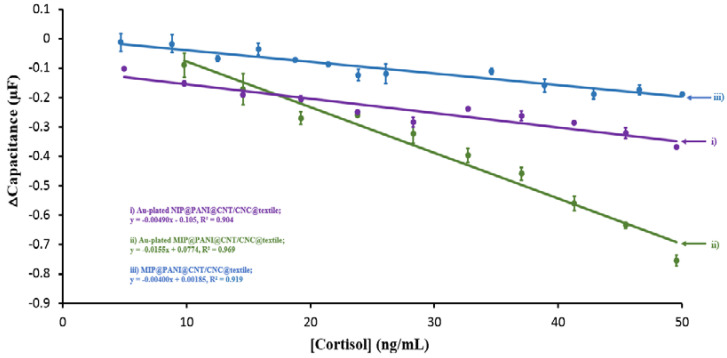
Linear calibration plots for Δcapacitance signal as function of cortisol concentration for (i) Au-plated NIP@PANI@CNT/CNC@textile, (ii) Au-plated MIP@PANI@CNT/CNC@textile, and (iii) MIP@PANI@CNT/CNC@textile cortisol sensor patches.

The MIP cortisol sensor and NIP patches were evaluated for their response to cortisol. Figure S5 outlines the representative voltammetric response of different patch variations (MIP@PANI@CNT/CNC@textile, Au-plated MIP@PANI@CNT/CNC@textile, and Au-plated NIP@PANI@CNT/CNC@textile) to phosphate buffer blank and cortisol. The Au-plated MIP@PANI@CNT/CNC@textile cortisol sensor patch displayed a significant response differential between the phosphate buffer blank and cortisol (Figure S5). Figure 6 shows the linear calibration plots for each cortisol sensor patch variation. To generate the Δcapacitance signal, the cathodic −0.5–0 V range was used for the MIP@PANI@CNT/CNC@textile cortisol sensors, while the cathodic 0.5–1.0 V range was used for Au-plated MIP and NIP@PANI@CNT/CNC@textile cortisol sensor patches (Figure 6). Addition of the Au plating to the MIP@PANI@CNT/CNC@textile cortisol sensor patch increased the calibration sensitivity from −0.00400 µF⋅mL/ng to −0.0155 µF⋅mL/ng (Figure 6). Additionally, the higher calibration sensitivity of the Au-plated MIP@PANI@CNT/CNC@textile cortisol sensor patch relative to the Au-plated NIP@PANI@CNT/CNC@textile patch (Figure 6) is indicative of enhanced selectivity from the cortisol-specific cavities within the MIP network [38]. Mechanistically, cortisol is captured in the cortisol-specific cavities of the MIP layer, preconcentrating it at the textile electrode [21]. CV application leads to a decrease in measured current with increasing cortisol concentration due to its electrically insulative properties [21]. The imprinting factor, calculated by dividing the calibration sensitivity of the Au-plated MIP@PANI@CNT/CNC@textile cortisol sensor by that of the Au-plated NIP@PANI@CNT/CNC@textile patch, was determined to be 3.16. Overall, the Au-plated NIP@PANI@CNT/CNC@textile patch demonstrates inferior performance compared to the Au-plated MIP@PANI@CNT/CNC@textile cortisol sensor patch, demonstrated by its lower calibration sensitivity. The low performance may be attributed to non-specific hydrophobic binding effects of cortisol. 

The LOD of cortisol using the Au-plated MIP@PANI@CNT/CNC@textile cortisol sensor patches was determined to be 8.00 ng/mL, well within the lower limits of the physiological range in human sweat [39,40]. The dynamic range for the AuNPs@MIP@PANI@CNT/CNC@textile cortisol sensor patch was 9.80–49.5 ng/mL, which again lies within the most common physiological cortisol range of 8–50 ng/mL [16]. However, continuous improvement of the sensor design is necessary to afford a much wider concentration range, which can be achieve by optimizing the surface area coverage of MIP layer on the PANI@CNT/CNC@textile patch. Compared to aptasensors reported in the literature, the dynamic range of the Au-plated MIP@PANI@CNT/CNC@textile cortisol sensor patch is superior, while its LOD is comparable to MIP and aptasensor based platforms (Table 2). However, cortisol immunosensors may have a much lower LOD [41], which is not useful for cortisol monitoring and mental health diagnostics, due to being outside of the typical 8–50 ng/mL physiological cortisol range [16]. Additionally, their lack of environmental stability makes their long-term use in real-time cortisol monitoring difficult. Compared to a PDMS-based sensor, the textile-based sensor lends better to wearability, sweat sampling and MIP layer stability [21,25]. In addition, the fabrication, cost, and scalability in production was found to be better in textile-based sensors. Compared to other cortisol sensors, the reported cortisol sensor patch is low-cost, easy to fabricate, and has a reasonable detection range and low LOD.

### 3.3. Evaluation of MIP@PANI@CNT/CNC@Textile Cortisol Sensor Selectivity

**Figure 7:**
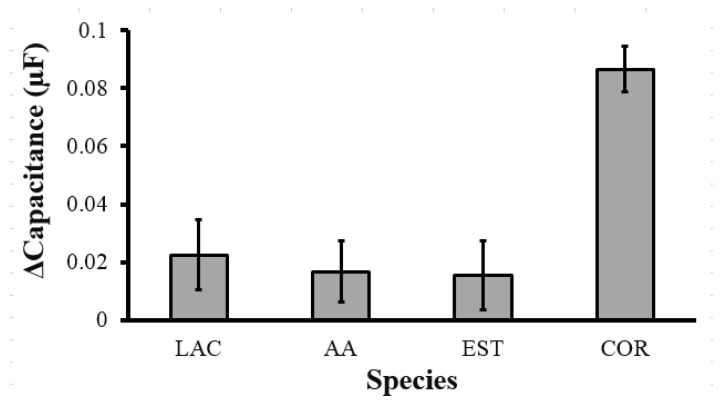
ΔCapacitance over the 0.5–1.0 V range obtained from analysis of 45 ng/mL lactate (LAC); 45 ng/mL ascorbic acid (AA); 45 ng/mL estriol (EST); and 45 ng/mL cortisol (COR) in 0.1 M phosphate buffer using an MIP@PANI@CNT/CNC@textile cortisol sensor patch.

To verify the selectivity of the MIP@PANI@CNT/CNC@textile cortisol sensor, the Δcapacitance response to lactate (LAC), ascorbic acid (AA), and estriol (EST) were compared to that of cortisol (COR). The CV current over the 0.5–1.0 V range (Figure S6) and the resulting Δcapacitance showed a general increase when each interfering compound was analyzed (Figure 7). Analysis of LAC, AA, and EST resulted in similar Δcapacitance increases of 0.02 ± 0.01, 0.02 ± 0.01, and 0.02 ± 0.01 µF, respectively (Figure 7). The largest Δcapacitance increase was generated from the analysis of cortisol, representing an increase of 0.087 ± 0.008 µF (Figure 7). This enhanced response to cortisol compared to the other interfering species confirms the selectivity of the MIP@PANI@CNT/CNC@textile cortisol sensor.

### 3.4. Evaluation of AuNPs@MIP@PANI@CNT/CNC@Textile Sensor for Sweat Analysis and Reusability Test

**Figure 8:**
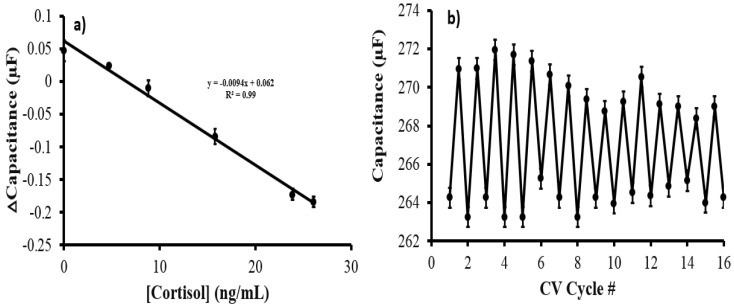
(**a**) ΔCapacitance vs. cortisol concentration standard addition calibration for sweat sample analysis using the AuNPs@MIP@PANI@CNT/CNC@textile cortisol sensor patch; and (**b**) Summative capacitance data vs. CV cycle number from the AuNPs@MIP@PANI@CNT/CNC@textile cortisol sensor patch reusability test.

The AuNPs@MIP@PANI@CNT/CNC@textile cortisol sensor patch was evaluated for electrochemical detection of cortisol in human sweat using the standard addition method. Generally, as the concentration of cortisol increases, the current within the −0.5–0 V range decreases, as shown in the voltammogram (Figure S7). Accordingly, the Δcapacitance vs. cortisol concentration standard addition calibration is shown in Figure 8a, with the capacitance signal generated from the current values averaged within the −0.5 to 0 V range. The cortisol concentration in the sweat sample was determined to be 19.7 ± 0.5 ng/mL, which is well within the typical physiological range [16,22,24,39].

Additionally, the AuNPs@MIP@PANI@CNT/CNC@textile cortisol sensor patch was evaluated for its reusability and long-term stability in storage. A 0.1 M phosphate buffer blank and a 20 ng/mL cortisol standard were analyzed by the same sensor patch once every 2 days over a 30-day period while being stored in room temperature conditions. Following each cortisol standard analysis, the AuNPs@MIP@PANI@CNT/CNC@textile cortisol sensor patch was electrochemically cleaned, as described in Section 2.2. The faradaic capacitance stayed relatively stable during the 30 days of storage, usage, and electrochemical cleaning (Figure 8b). Further, based on three different sensors’ data, the intra-batch percent %RSD was determined to be 2.16%. Based on these results, the stability and reusability of the AuNPs@MIP@PANI@CNT/CNC@textile cortisol sensor patch is demonstrated.

## 4. Conclusions

Cortisol is an important biomarker in human sweat related to physiological stress, anxiety, and depression. This manuscript demonstrates an inexpensive and wearable AuNP-coated, cortisol-imprinted poly(GMA-co-EGDMA) MIP-based capacitive textile sensor patch (AuNPs@MIP@PANI@CNT/CNC@textile) with an integrated screen printed three-electrode system, capable of selectively detecting cortisol in human sweat. The Au-enhanced, cortisol-imprinted MIP textile sensor responds linearly to 9.8–49.5 ng/mL of cortisol, with a LOD of 8.00 ng/mL. The cortisol sensor patch was robust and reusable, and could accurately detect cortisol for over 15 detection cycles performed over a 30-day period with high precision (%RSD = 2.2%). The softness and flexibility of the cotton textile substrate enhances the wearing comfort when attached to human skin. Lastly, the high liquid absorbent property of the cotton textile can soak more sweat, thus affording more accurate results. The cortisol sensor sensitivity may however be improved by use of chemical vapor deposition (CVD) of gold rather than the drop casting of the AuNPs solution, an approach for future study.

## Figures and Tables

**Table 1:** Electroactive surface areas and electron transfer resistances (R_ct_) for the various patch designs.

Sensor Type	Electroactive Surface Area (cm^2^)	R_ct_ (×10^3^ Ω)
PANI@CNT/CNC@textile	0.0120	0.703
AuNPs@MIP@PANI@CNT/CNC@textile	0.0264	1.59
AuNPs@NIP@PANI@CNT/CNC@textile	0.00497	-
NIP@PANI@CNT/CNC@textile	0.00469	3.16
MIP@PANI@CNT/CNC@textile	0.00529	2.23
CNT/CNC@textile	0.00114	29.2

**Table 2:** Literature comparison of cortisol sensor performance metrics to the reported Au-plated MIP@PANI@CNT/CNC@textile cortisol sensor patch.

Sensor Type	Recognition Surface	Concentration Range (ng/mL)	LOD (ng/mL)	Reference
ZnO nanorod-integrated flexible carbon fibers	ZnONRs/CCY immunosensing platform	1.0 × 10^−6^–1.0 × 10^3^	9.8 × 10^−8^	Madhu et al., 2020 [41]
Cortisol/insulin dual electrochemical immunosensor microchip	Alkaline phosphatase (ALP)-labeled competitive immunoassay	0–250	13.4	Vargas et al., 2020 [26]
Aptamer-based lateral flow biosensor	Cortisol aptamer	0.5–15	0.37	Dalirirad et al., 2020 [27]
Cortisol-specific DNA aptamer@CNT/CNC@PDMS sensor	Cortisol-specific DNA aptamer	2.5–35	1.8	Mugo et al., 2021 [25]
Cortisol MIP@CNT/CNC@ PDMS sensor	Cortisol-imprinted poly(GMA-co-EGDMA)	10–66	2.0 ± 0.4	Mugo et al., 2020 [21]
Graphene-based capacitive sensor	Carboxylate-rich pyrrole-derivative grafting	<10	-	Torrente-Rodríguez et al., 2020 [40]
AuNP-basedcortisol sensor	Room-temperature plasma sintering technique	5.0 × 10^−4^–30	0.12	Sonawane et al., 2021 [42]
Cortisol-selective MIPs	MIP technique	36.2–362	-	Daniels et al., 2021 [43]
Au-plated MIP@PANI@CNT/CNC@textile cortisol sensor patch	Cortisol imprinted poly(GMA-co-EGDMA) with Au enhancement	9.80–49.5	8.00	This work

## Data Availability

All relevant data is included in the manuscript and in the supporting information.

## Supplementary Materials

The following supporting information can be downloaded at: https://www.mdpi.com/article/10.3390/bios12100854/s1, Figure S1. Overlaid CVs taken during the (i) 2nd scan; and (ii) 15th scan of the first electrochemical cleaning cycle; and (iii) 2nd; and (iv) 15th scan of the last electrochemical cleaning cycle using the MIP@PANI@CNT/CNC@textile cortisol sensor patch. Figure S2. Schematic for the LbL assembly of the AuNPs@MIP@PANI@CNT/CNC@textile cortisol sensor patch. Figure S3. Camera image of the AuNPs@MIP@PANI@CNT/CNC@textile taken (a) before; (b) during; and (c) after crumpling with tweezers. Figure S4. EIS circuit fitting for different variations of cortisol sensor patches. Figure S5. Overlaid CVs for different cortisol sensor patch variations obtained from analysis of 0.1 M PBS blank and 26.08 or 28.3 ng/mL cortisol (COR): (i–ii) MIP@PANI@CNT/CNC@textile; (iii–iv) Au-plated MIP@PANI@CNT/CNC@textile; and (v–vi) Au-plated NIP@PANI@CNT/CNC@textile. Figure S6. Overlaid CVs range obtained from analysis of (i) 45 ng/mL lactate (LAC); (ii) 45 ng/mL ascorbic acid (AA); (iii) 45 ng/mL estriol (EST); and (iv) 45 ng/mL cortisol (COR) in 0.1 M phosphate buffer using an MIP@PANI@CNT/CNC@textile cortisol sensor patch. Figure S7. Overlaid voltammograms from the standard addition calibration for sweat sample analysis using the AuNPs@MIP@PANI@CNT/CNC@textile cortisol sensor patch.
