# Supplementary material for: A Wearable, Textile-Based Polyacrylate Imprinted Electrochemical Sensor for Cortisol Detection in Sweat
Source: Biosensors (Basel). 2022 Oct 10;12(10):854. doi: 10.3390/bios12100854 (PMC9599184; doi:10.3390/bios12100854)
Supplement: Supplementary file 1 [file biosensors-12-00854-s001.zip › biosensors-1938025-supplementary.pdf]

A Wearable, Textile-based Polyacrylate Imprinted Electrochemical Sensor for Cortisol Detection in Sweat

Samuel M. Mugo [1*], Weihao Lu [1], and Scott Robertson [1]

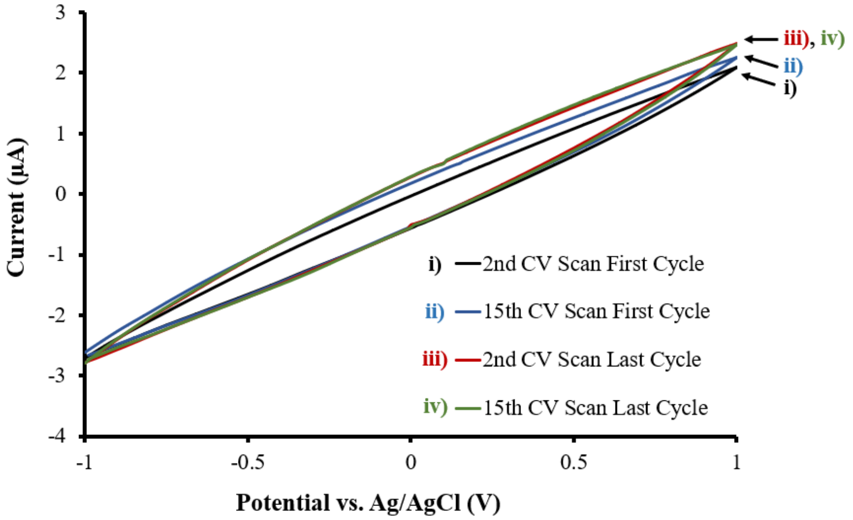

Figure S1. Overlaid CVs taken during the i) 2nd scan; and ii) 15th scan of the first electrochemical cleaning cycle; and iii) 2nd; and iv) 15th scan of the last electrochemical cleaning cycle using the MIP@PANI@CNT/CNC@textile cortisol sensor patch.

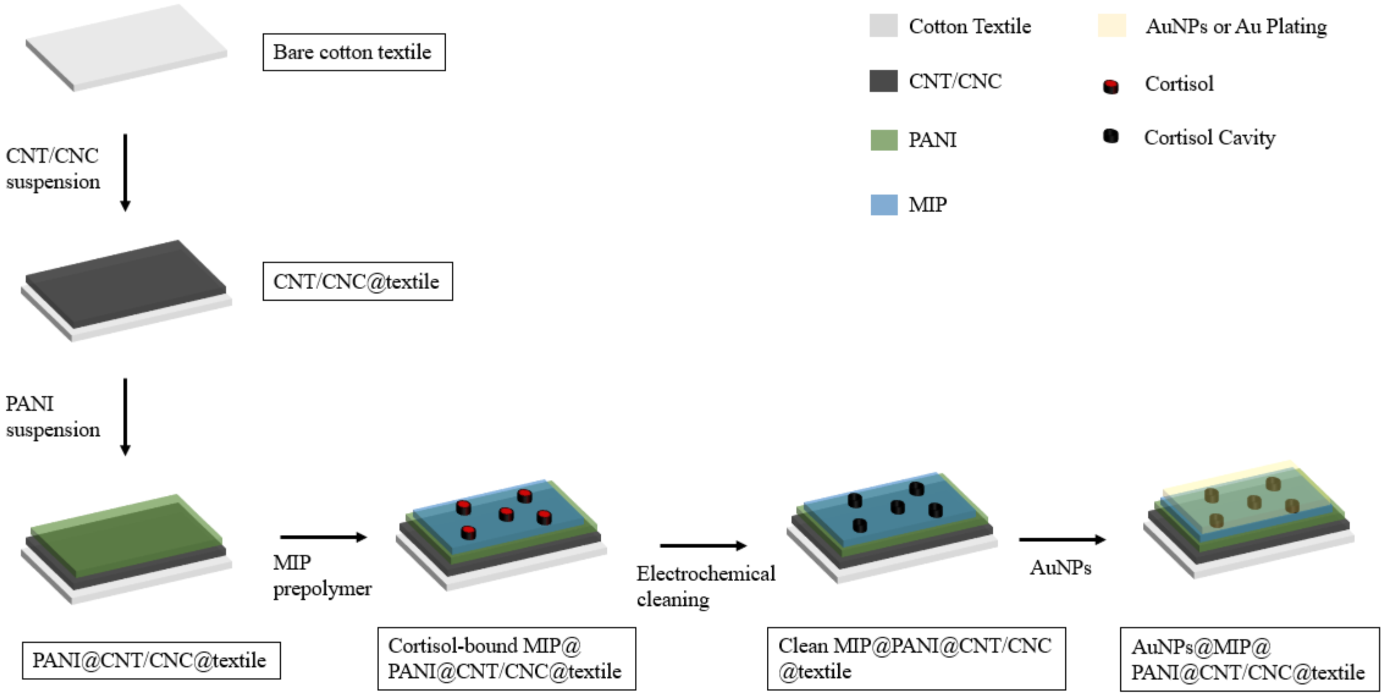

Figure S2. Schematic for the LbL assembly of the AuNPs@MIP@PANI@CNT/CNC@textile cortisol sensor patch.

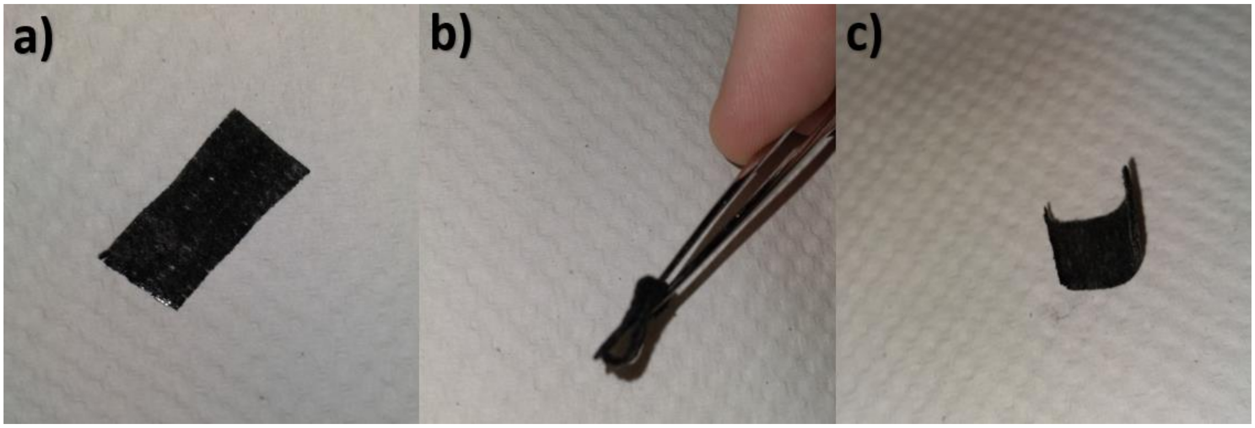

Figure S3. Camera image of the AuNPs@MIP@PANI@CNT/CNC@textile taken **a)** before; **b)** during; and **c)** after crumpling with tweezers.

Figure S4. EIS circuit fitting for different variations of cortisol sensor patches.

a) CNT/CNC@textile

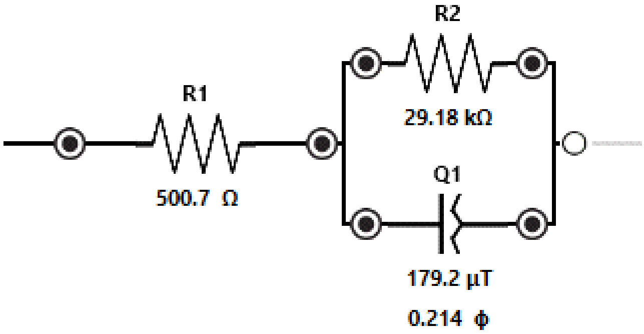

b) PANI@CNT/CNC@textile

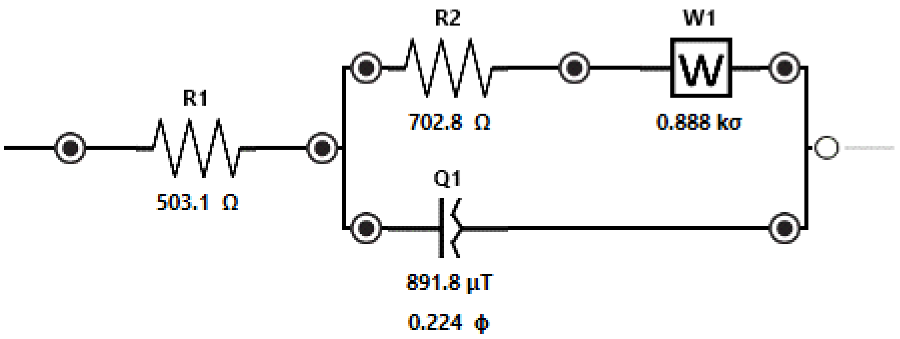

c) MIP@PANI@CNT/CNC@textile

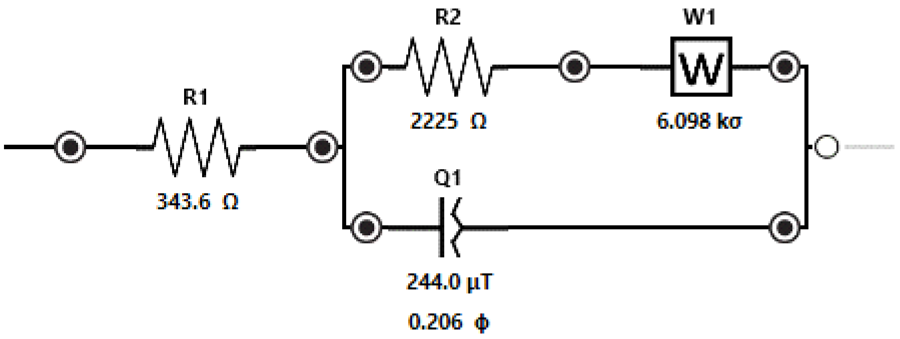

d) NIP@PANI@CNT/CNC@textile

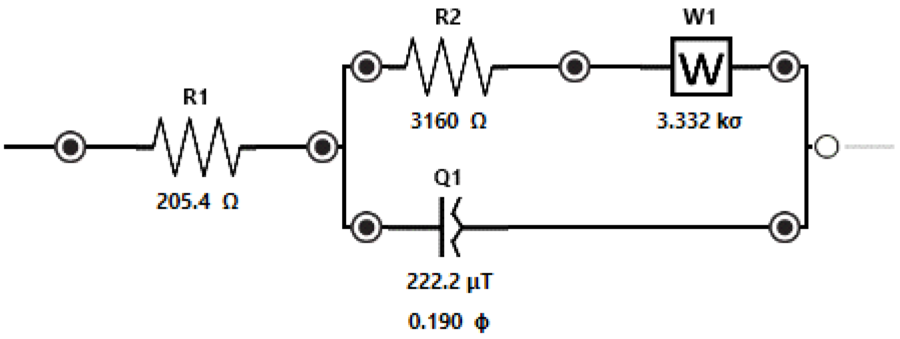

e) AuNPs@MIP@PANI@CNT/CNC@textile

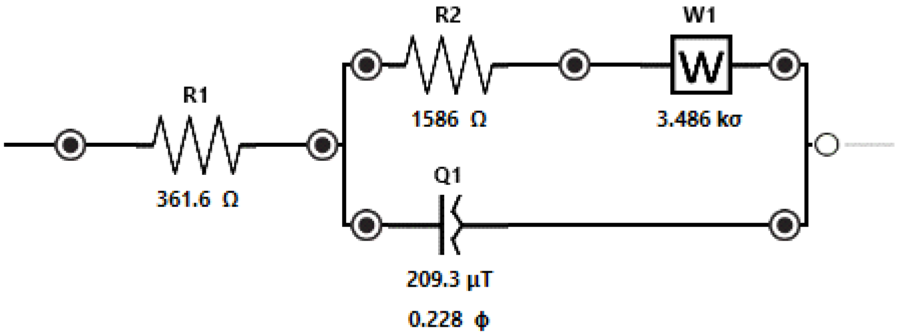

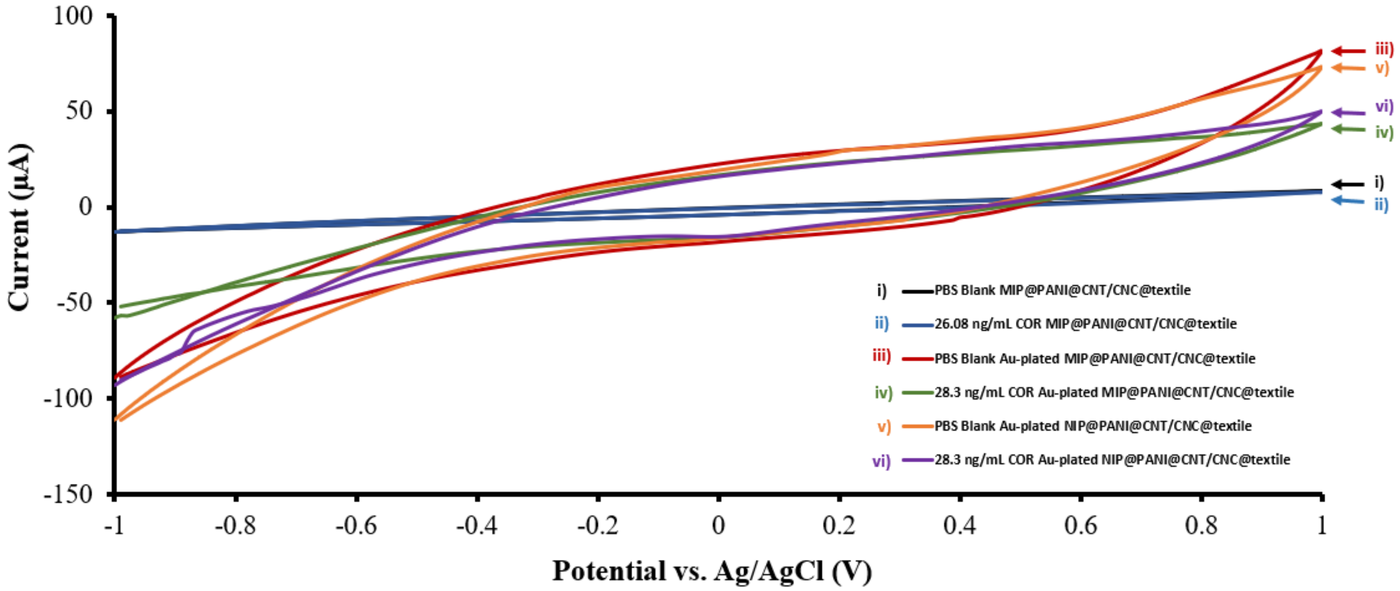

Figure S5. Overlaid CVs for different cortisol sensor patch variations obtained from analysis of 0.1 M PBS blank and 26.08 or 28.3 ng/mL cortisol (COR): **i), ii)** MIP@PANI@CNT/CNC@textile; **iii), iv)** Au-plated MIP@PANI@CNT/CNC@textile; and **v), vi)** Au-plated NIP@PANI@CNT/CNC@textile.

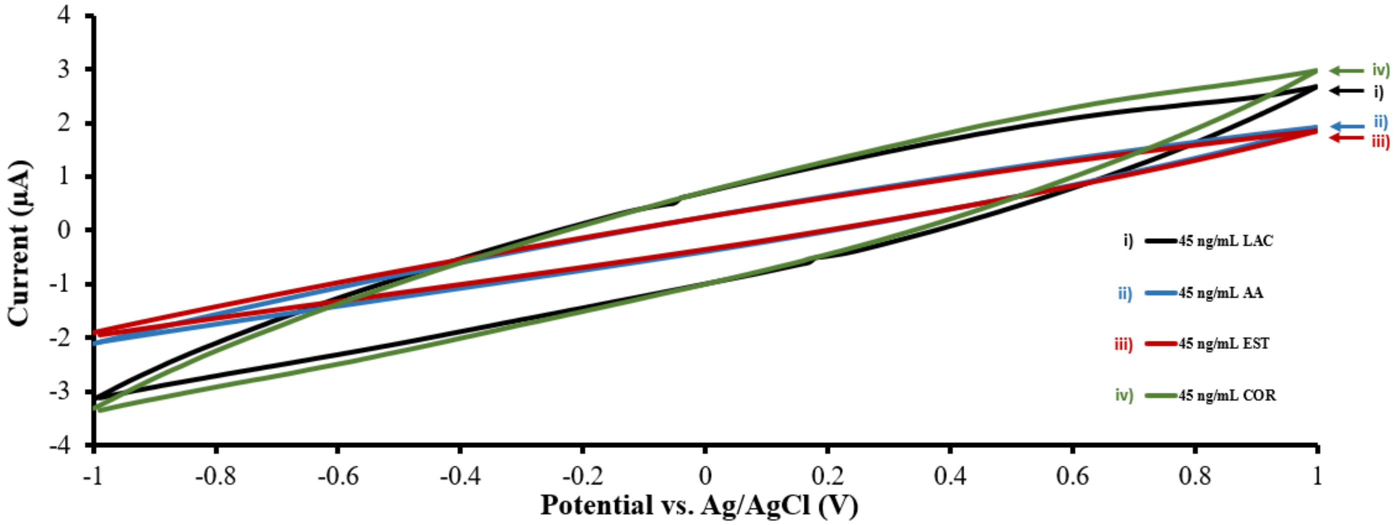

Figure S6. Overlaid CVs range obtained from analysis of i) 45 ng/mL lactate (LAC); ii) 45 ng/mL ascorbic acid (AA); iii) 45 ng/mL estriol (EST); and iv) 45 ng/mL cortisol (COR) in 0.1 M phosphate buffer using an MIP@PANI@CNT/CNC@textile cortisol sensor patch.

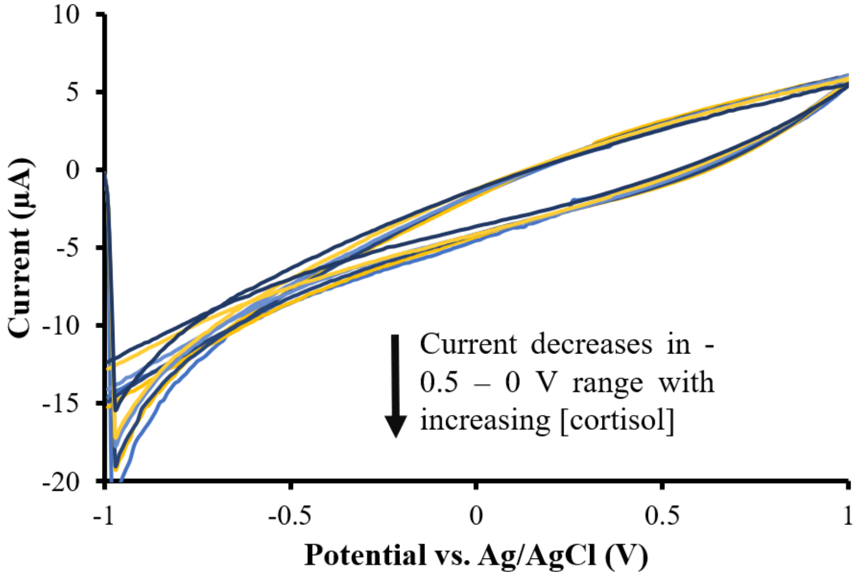

Figure S7. Overlaid voltammograms from the standard addition calibration for sweat sample analysis using the AuNPs@MIP@PANI@CNT/CNC@textile cortisol sensor patch.
